# Sugar-sweetened beverage but not diluted cloudy apple juice consumption induces post-prandial endotoxemia in healthy adults

**DOI:** 10.1038/s41538-024-00283-w

**Published:** 2024-06-21

**Authors:** Raphaela Staltner, Sarah Valder, Maximilian F. Wodak, Magdalena Köpsel, Volker Herdegen, Tuba Esatbeyoglu, Tihomir Kostov, Patrick Diel, Ina Bergheim

**Affiliations:** 1 Department of Nutritional Sciences, Molecular Nutritional Science, University of Vienna, Vienna, Austria; 2 Institute of Cardiology and Sports, Section Molecular and Cellular Medicine, German Sport University Cologne, Cologne, Germany; 3 Department of Molecular Food Chemistry and Food Development, Institute of Food Science and Human Nutrition, Gottfried Wilhelm Leibniz University Hannover, Hannover, Germany; 4 Research and Innovation, Eckes-Granini Group GmbH, Nieder-Olm, Germany

**Keywords:** Gastroenterology, Diseases

## Abstract

Sugar beverages are discussed as critical in the development of metabolic endotoxemia. Here, employing a cross-over design study we assessed the effect of diluted cloudy apple juice (AJ), an iso-caloric and -sweetened placebo (P), or water (W) on post-prandial endotoxemia in healthy, normal weight adults. After obtaining fasting blood, 19 healthy men and women consumed 500 mL AJ, P, or W in a randomized order and blood was taken 120 and 180 min later. Caco-2 cells were incubated with the beverages. Markers of intestinal barrier function were assessed. The intake of P but not of AJ or W was associated with a significant increase in TLR2 ligands and bacterial endotoxin in serum after 120 min and 180 min, respectively. P but not AJ significantly increased bacterial toxin permeation in Caco-2 cells. Our results suggest that the effects of sugar-sweetened beverages on markers of intestinal barrier function markedly differ from those of fruit juices.

## Introduction

Besides general overnutrition, sugar intake and herein especially that derived through the intake of sugar-sweetened beverages, has gained particular attention as a contributor to the development of overweight and obesity as well as associated cardiometabolic diseases throughout the last decades. For instance, results of a recent meta-analysis systematically analyzing data obtained in 27 longitudinal studies suggest that the intake of sugar-sweetened beverages increases the risk of type 2 diabetes, obesity, coronary heart disease and stroke in adults^1^. Findings of a recent cross-sectional study further suggest that fructose consumed through sugar-sweetened beverages may lead to adverse effects on cardiometabolic biomarkers and pro-inflammatory markers^2^. In contrast, in most studies similar relations were not found for low to modest intakes of 100% fruit juice^3–8^. The somewhat conflicting results where a high intake of fruit juice was related to the increased risk of developing overweight^9^ and cardiometabolic diseases^10^ have recently been discussed to be also dependent on how fruit juice is defined^11^. These data further suggest that more well-defined, controlled studies employing clearly defined beverages are needed.

Elevated bacterial endotoxin levels frequently found in settings of obesity and metabolic diseases having been related to impairments of intestinal barrier function and alterations of intestinal microbiota composition have repeatedly been referred to as ‘metabolic endotoxemia´ in recent years (for overview also see ref. ^12^). These alterations are discussed to be critical in the onset and also the progression of many metabolic diseases (for an overview see ref. ^13^). Indeed, elevated levels of bacterial endotoxin but also of wall compounds found in Gram-positive bacteria like lipoteichoic acid (LTA) and peptidoglycan and an activation of their receptor-dependent signaling pathways, e.g., signaling pathways regulated by Toll-like receptor 4 and 2 (TLR4 and TLR2), have been shown to be related to the development of obesity and metabolic alterations in humans^14–16^. Furthermore, results of our own group but also others suggest that both an inhibition of TLR4 and TLR2 signaling through specific inhibitors or genetic deletion in mice is associated with a protection from the development of metabolic diseases like metabolic dysfunction-associated steatotic liver disease (MASLD) and type 2 diabetes^17–19^. It also has been shown that even the acute or short-term intake of specific foods or food compounds like saturated fats or fructose and sucrose^20–22^ may increase bacterial endotoxin levels in humans thereby adding to the development of the so-called post-prandial endotoxemia^23^. However, whether sugars consumed in their natural matrix like fruits or fruit juices affect intestinal barrier function and translocation of bacterial wall compounds has not yet been clarified in humans. Indeed, contrasting most soft drinks, fruit juices contain a variety of secondary plant metabolites that are absorbed in the intestine and are also metabolized by intestinal cells. For instance, results of in vitro and ex vivo studies suggest that polyphenols like those found in apple juice are absorbed and metabolized^24,25^ by intestinal cells and may thereby possess anti-oxidant and anti-inflammatory properties^26^ and even decrease DNA damage and hyperproliferation^27,28^.

Starting from this background, the aim of the present placebo-controlled cross-over design study was to compare the effects of the acute intake of 500 mL diluted cloudy apple juice (AJ) with an iso-caloric sugar-sweetened placebo beverage (P) lacking secondary plant metabolites and the intake of water (W) on post-prandial endotoxemia and markers of intestinal barrier function in healthy young adults. Moreover, effects of diluted cloudy apple juice on bacterial toxin permeation were assessed in differentiated Caco-2 cells grown in a trans-well system mimicking intestinal barrier in small intestine.

## Results

### Baseline characteristics

In total, 26 normal weight healthy women and men aged 18–35 years were enrolled in the study. Nineteen participants finished the study and were analyzed (see Supplementary Figure 1). Characteristics of participants including anthropometry and routine laboratory parameters are summarized in Table 1. Nutritional intake of participants before the iso-caloric standardization to a diet recommended by the German, Austrian, and Swiss (DACH) nutrition societies is shown in Table 2. During the standardization mean intake of carbohydrates had to be increased significantly (~+12%), whereas protein intake of participants had to be lowered by 0.7 g/kg body weight in order to meet the recommendations of an appropriate daily intake (0.8 g/kg body weight/day). Total fat intake remained unchanged. In line with previous findings of our group^20,21^ the nutritional standardization was associated with a significant decrease of bacterial endotoxin levels in peripheral blood (Table 2).

**Table 1:** Characteristics of healthy human male and female participants

Parameters	Baseline
Gender (m/f)	15/4
Age (years)	26 ± 4
BMI (kg/m^2^)	22.3 ± 0.4
Blood glucose (mg/dL)	79.6 ± 1.2
Triglycerides (mg/dL)	87.6 ± 6.5

Values are means ± SEM, *n* = 19.

*BMI* body mass index.

**Table 2:** Results of 24-h recall and standardization of nutrition

Nutrients	24-h recall	Standardization
Energy (kcal/d)	2566 ± 154	2564 ± 136
Carbohydrates (E %) (>50 E %)^a^	47.2 ± 2.2	59.3 ± 0.2*
Protein (g/kg body weight/day) (0.8 g/kg body weight/day)^a^	1.5 ± 0.1	0.8 ± 0.0*
Fat (E %) (30 E %)^a,b^	32.8 ± 2.5	31.5 ± 0.1
Bacterial endotoxin (OD)	0.28 ± 0.02	0.24 ± 0.02*

Values are means ± SEM, *n* = 19.

^a^Recommendations where applicable.

^b^Higher percentages possible with physical activity level >1.7.

*p < 0.05.

### Effect of the acute intake of AJ, P, or W on markers of intestinal permeability

**Fig. 1:**
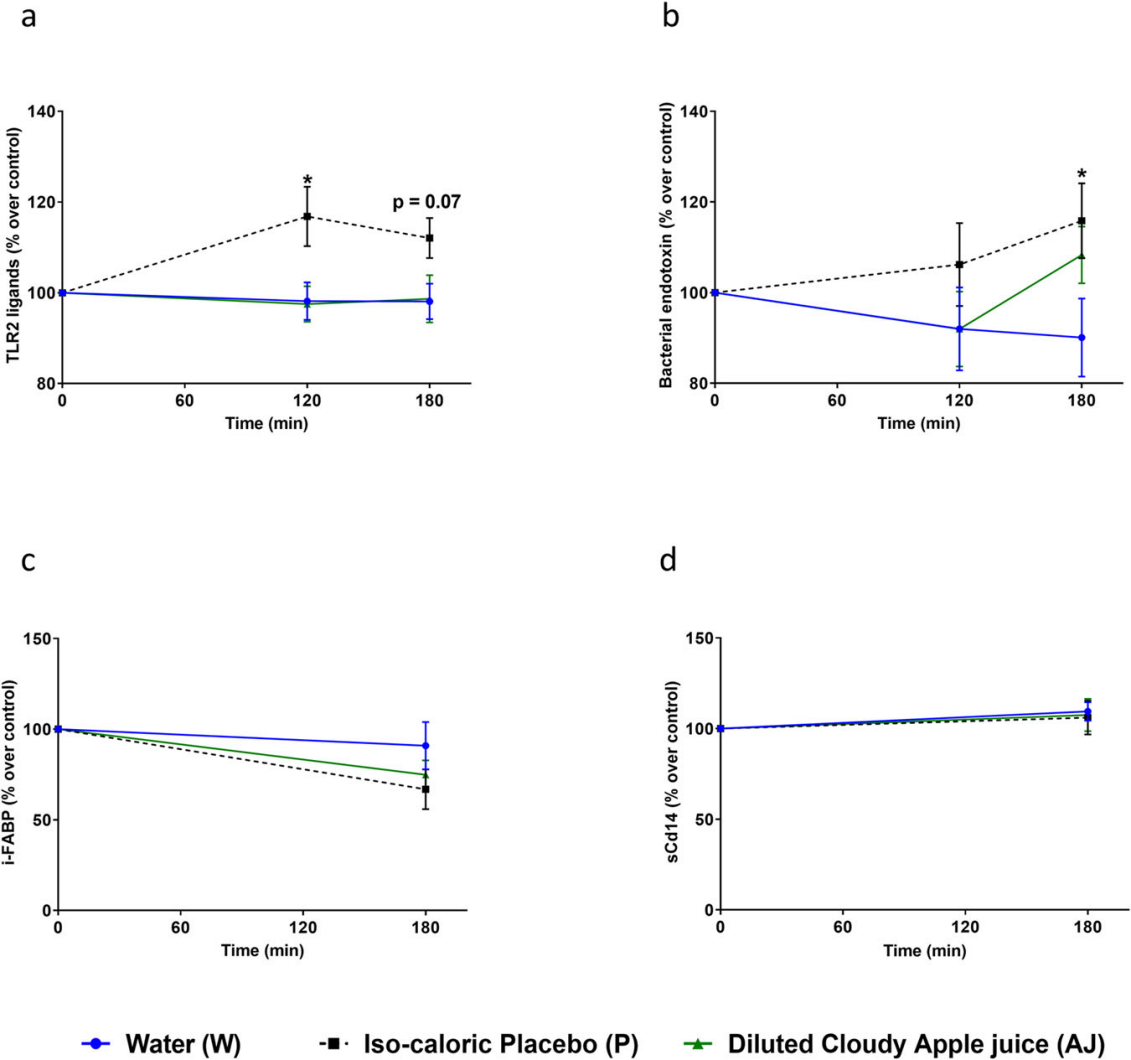
Effect of water (W), iso-caloric and iso-sweet placebo (P), and diluted cloudy apple juice (AJ) in serum of participants 120 and 180 min after the consumption of the respective beverage. **a** TLR2 ligands and **b** bacterial endotoxin levels in plasma. Effect of the different beverages on **c** i-FABP as well as on **d** sCd14 in serum of participants 180 min after consumption of the beverage. For (**a**): *p < 0.05 placebo vs water after 120 min and *p* = 0.07 placebo vs water after 180 min. For (**b**): *p < 0.05 placebo vs water after 180 min. Values are means ± SEM, *n* = 19. TLR Toll-like receptor, sCd14 soluble cluster of differentiation 14, i-FABP intestinal fatty acid binding protein.

Consumption of the P, containing a mix of sucrose and fructose as well as glucose being identical to that found in the AJ resulted in a significant and by trend increase of TLR2 ligands 120 min after ingestion (*p* < 0.05) and 180 min after ingestion (*p* = 0.07) compared to (W). No increase of TLR2 ligands in serum was found after subjects ingested the AJ or W (Fig. 1a). The intake of P resulted in a significant increase of bacterial endotoxin 180 min after ingestion compared to water (Fig. 1b). Consumption of the AJ and W had no effect on bacterial endotoxin levels in serum. Also, after the ingestion of either of the beverages, no changes were found in intestinal fatty acid binding protein (i-FABP) or soluble Cluster of differentiation 14 (sCd14) protein levels in serum (Fig. 1c, d), both having been suggested before to be indicative of alterations in intestinal integrity^29,30^.

### Effect of AJ and P on bacterial toxin permeation and fructose levels in cell culture medium of differentiated Caco-2 cells

**Fig. 2:**
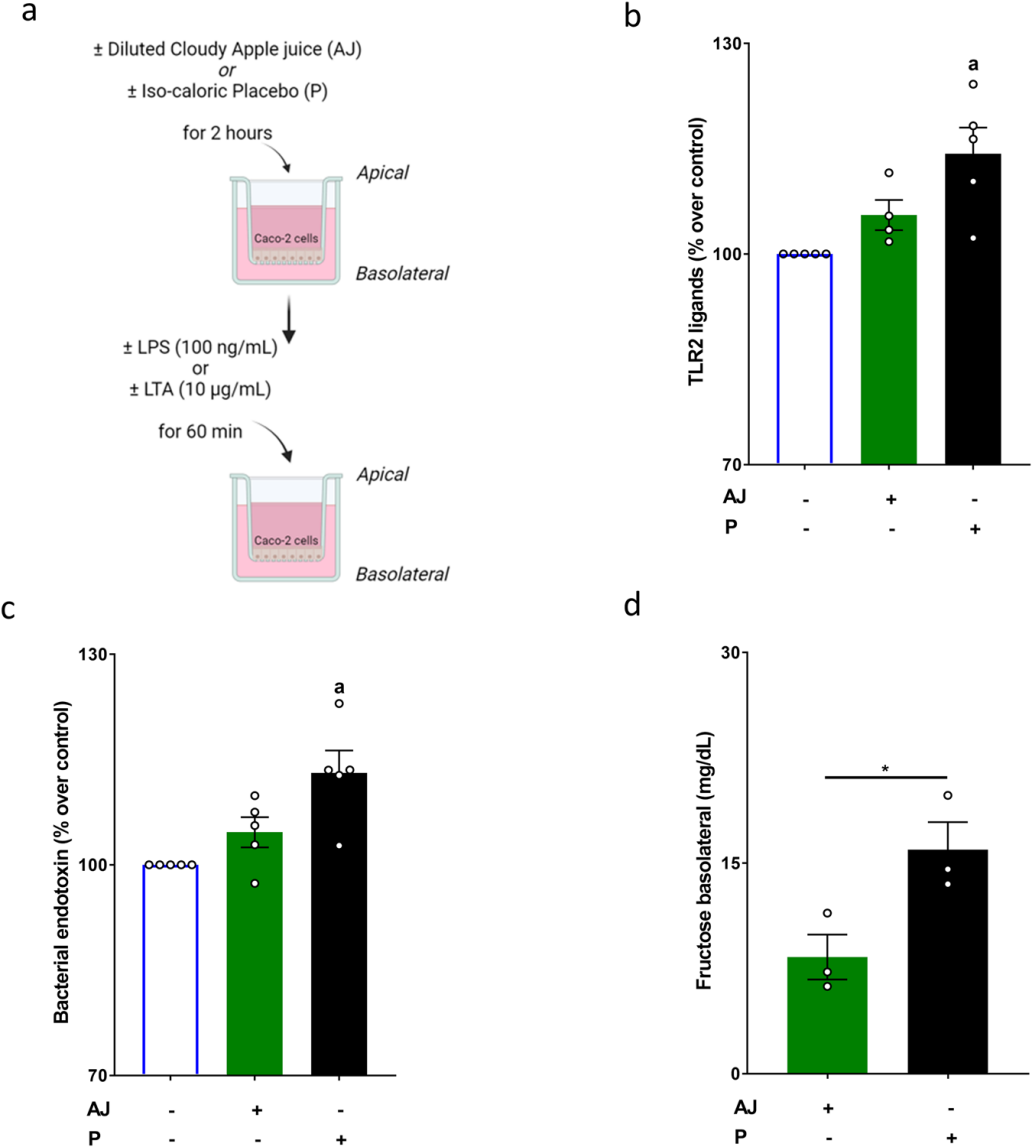
Effect of the iso-caloric and iso-sweet placebo (P), and diluted cloudy apple juice (AJ) on permeability of bacterial toxins in differentiated Caco-2 cells grown in a trans-well system. **a** Graphical illustration of experimental set-up of the cell culture experiment. Measurement of **b** TLR2 and **c** bacterial endotoxin levels in the basolateral compartment of the trans-well model. **d** Fructose concentration in the basolateral compartment of the trans-well model. *p < 0.05. Values are means ± SEM, *n* = 3–5. TLR Toll-like receptor. Graphical illustration (**a**) was created with BioRender.com.

In line with the findings in vivo, the 3-h challenge of differentiated Caco-2 cells grown on trans-wells with P resulted in a significantly higher translocation of LTA and lipopolysaccharide (LPS) when compared to naive cells. Specifically, LTA and bacterial endotoxin levels, respectively, were ~1.14-fold higher in the basolateral compartment of the well-system when cells were challenged with P compared to naive cells and those exposed to the AJ. In contrast, both LTA and bacterial endotoxin concentrations were at the level of controls in the basolateral compartment of Caco-2 cells exposed to AJ (Fig. 2b, c). Moreover, while the same concentrations of sugars were added to the apical compartment of the trans-well system, fructose concentration in the basolateral compartment of the trans-well system was significantly lower in cells exposed to the AJ when compared to those exposed to the P (Fig. 2d).

## Discussion

Besides adding to overnutrition and subsequently an expansion of fatty tissue it has also been discussed that sugars ingested through beverages may have more direct effects on health e.g., through altering intestinal microbiota composition and/or intestinal barrier function in small intestine (for overview see refs. ^31,32^). Despite intense research efforts, the question if fruit juices containing polyphenols and other secondary plant metabolites have similar adverse effects on body weight, metabolic health, and the intestinal barrier as those reported for sugar-containing drinks has not yet been clarified. Here, after nutritionally standardizing participants to a healthy diet following the recommendations of the German, Austrian, and Swiss nutrition societies, study subjects consumed once either 500 mL of diluted cloudy apple juice or an iso-sweetened and iso-caloric as well as flavored placebo or plain water. Diluted cloudy apple juice was selected as study beverage, as in German speaking countries cloudy apple juice is a rather commonly consumed fruit juice^33^. Also, diluted juices e.g., apple juice, is recommended in the regeneration after physical training^34^. In line with previous studies of our group^20,21^, the standardization of the nutritional intake of participants to a ‘healthy´ recommended diet was related with a significant decrease of bacterial endotoxin levels in peripheral blood. It remains to be determined, if this decrease was related to the decrease of total protein and increase of total carbohydrate intake or the overall change in dietary pattern (e.g., increased intake of fruits and vegetable, wholegrain products, reduction of sweets and candy). It has been shown before that even in healthy individuals 3-hydroxy fatty acids levels (a lipid in the outer membrane of Gram-negative bacteria and used as proxy of bacterial endotoxin), are lower when they regularly follow a diet considered healthy e.g., Mediterranean or prudent diet compared to those following for instance an ‘unhealthy´ diet^35^. The intake of the placebo, but not of the diluted cloudy apple juice or plain water was associated with an increase of bacterial endotoxin and of TLR2 ligand levels in peripheral blood. Neither the concentration of i-FABP protein nor sCd14 in serum was altered after the consumption of either of the beverages. Results of the cell culture experiments of our own group but also other groups have suggested that upon a stimulation of cell ‘stressors´ like sucrose and oleic acid, i-FABP protein levels in cell culture medium of differentiated Caco-2 cells may increase within 2–3 h of the challenge^21,36^. Also, in vitro studies employing isolated peripheral blood mononuclear cells or monocytes have reported increases in sCd14 within 6 h after challenges with LTA and LPS, respectively^37,38^. It could be that in the present study, the time of exposure and the ‘stressors´ were not sufficient e.g., too low in concentration to increase concentration of either protein in peripheral blood. Still, our results are in line with previous results of our own group and those of others showing that the acute ingestion of beverages sweetened with sucrose may result in an increase of bacterial endotoxin levels in peripheral blood while the intake of orange juice may not lead to alterations alike^21,39^. Results of previous human and animal studies further suggest that this may be related to the presence of fructose found in sucrose rather than glucose^20,40^.

In line with the findings in vivo, only the challenge of differentiated Caco-2 cells with the placebo beverage (P) resulted in an increased permeation of the bacterial toxins. It has been shown before that upon being challenged with sugars and herein especially fructose, permeability of differentiated Caco-2 cells can be altered^41,42^. In animal and in cell culture experiments it has been demonstrated that polyphenols like those found in (cloudy) apple juice can diminish mucosa damage-related oxidative stress^43^. Also, they may induce the expression of mucins as well as increase protein levels of tight junction proteins^44–46^. Furthermore, pectin which at low levels is also found in cloudy apple juice (concentration in the diluted cloudy apple juice used in the present study 68 mg/L) has been suggested to dampen LPS-induced immune responses when LPS is applied intraperitoneally^47,48^. It has been shown in animal models and in in vitro experiments that pectin derived from citrus plant and papaya may interact with pattern recognition receptors (PAMPs) and may thereby diminish their response to challenges with bacterial toxins^49^. Also, when consumed regularly at higher concentration, pectin may also affect intestinal barrier function^50–52^. Moreover, it has been suggested that pectin gel particles may bind bacterial endotoxin^53^. It also has been shown that secondary plant compounds like apigenin but also catechins can diminish fructose uptake through interfering with GLUT5 expression^54^. Somewhat in line with these findings, in the present study, fructose concentration in the basolateral compartment of the trans-well system was markedly lower when compared to that found when cells were challenged with the placebo. However, if fructose uptake was diminished or the sugar was ‘further’ metabolized by the cells needs to be determined in future studies. Also, if fructose uptake/metabolism in intestinal cells was altered in vivo and in vitro remains to be determined.

In summary, our results suggest that the acute intake of sucrose and fructose through soft drinks in the absence of a fruit matrix like that found in apple juice may result in post-prandial endotoxemia in healthy young adults. Our results further suggest that compounds found in fruit matrices may alter physiological effects of sugars consumed in beverages. Further studies are needed to determine which compounds are critical herein as well as their mode of action. Also, it remains to be determined if these effects are also found, when diluted or plain apple juice is consumed for an extended period of time or at higher concentrations.

There are some limitations in our study that should be considered when interpreting the data. One limitation of our study is the focus on young healthy adults. Results may be different in another age group or in overweight subjects or subjects with metabolic abnormalities e.g., type 2 diabetes or MASLD. Future studies will be needed to assess this. Also, in the present study subjects only consumed the different beverages once and measurements were terminated after 180 min. Accordingly, no estimation regarding later time points or long-term effects of an intake of diluted or plain cloudy apple juice as well as of the placebo being at least partly similar to a sugar-sweetened soft drink on intestinal barrier function can be made. Also, to increase compliance, in the present study the test beverages were served along with a light breakfast consisting of 60 g oatmeal, 5 g honey prepared with water and a banana. It cannot be ruled out, that the banana may also have alleviated some of the adverse effects of fructose. Another limitation of our study is that we only assessed intestinal barrier function through indirect measures e.g., through assessing bacterial endotoxin and TLR2 ligands. However, direct tests to assess intestinal permeability like a lactulose-mannitol test or a xylose test require the ingestion of an additional beverage and a urine collection over several hours. As the study protocol was rather rigid and participants had to consume a large amount of liquid in a rather short period of time on the day of study, these additional tests would have resulted in a lower compliance and therefore in a higher drop-out rate.

In summary, our results suggest that contrasting the effects of an acute consumption of a sugar-sweetened beverage, the acute intake of diluted cloudy apple juice in physiological amounts e.g., 500 mL, has no effect on markers of intestinal barrier function in healthy young adults. If the repeated intake of diluted or undiluted cloudy apple juice over an extended period of time or in larger quantities also has no effect on intestinal barrier and if similar results are also found in individuals with health impairments, e.g., metabolic abnormalities remain to be determined. Results of our study further bolster the hypothesis that health effects of sugars and especially sucrose and/ or fructose may depend on the matrix e.g., whether they are consumed isolated or in their natural matrix like a whole fruit or a whole fruit juice. Indeed, results of the present in vitro experiments suggest that cellular responses to sugar may largely differ when the fruit matrix and its bioactive compounds e.g., polyphenols are present. Further studies are needed to determine underlying mechanisms and to identify specific compounds involved in these divergent health effects of whole fruit juice and sugars.

## Methods

### Study beverages

To match commercially available diluted apple juices, cloudy apple juice (orchard apple juice from concentrate, South of Germany) and demineralized water were blended to obtain a beverage with 60% fruit content (AJ). The iso-caloric and polyphenol-free placebo beverage (P) was prepared according to the analytical parameters of the study drink AJ by adding 13.4 g/L glucose, 35.0 g/L fructose, and 9.1 g/L sucrose (see Table 3). The latter drink was specifically mixed for the present study and adjusted to sugar content, which was lower than in most commercially available soft drinks (commercially available sugar-sweetened beverage ~100 g/L vs. ~58 g/L total sugar in the present study). Furthermore, both beverages contain sorbitol, malic acid, potassium, and apple aroma at similar levels (all analytical details see Table 4 and Supplementary Table 1). For the methods employed for analysis of the study drinks, please see below analytical details of study beverages. Both beverages were prepared according to good manufacturing practice, hot-filled into clear 0.5 L glass bottles and cooled back to 20 °C within ~15 min. The bottles were stored at room temperature until further use. Polyphenol content of apple juice was assessed as described in detail by the Folin-Ciocalteau^55^ before. A commercially available water (Volvic) was used as additional control (W).

**Table 3:** Composition of the test beverages

Ingredients	Water (W)	Placebo (P)	Diluted cloudy apple juice (AJ)
Glucose (g/L)	–	13.4	13.4
Fructose (g/L)	–	35.0	35.0
Sucrose (g/L)	–	9.1	9.1
Total sugar (g/L)	–	57.5	57.5
Apple flavor (mL/L)	–	4.7	4.7
Sorbitol (g/L)	–	2.2	2.2
Malic Acid (g/L)	–	3.4	3.4
Potassium (g/L)	–	0.6	0.6
Total Polyphenols (mg/L)	–	–	550

Values are means, *n* = 3.

**Table 4:** Characterization of the cloudy apple juice and two study drinks

		Study beverages	
Parameters	100% Apple juice	Diluted cloudy apple juice (AJ)	Placebo (P)
Density (g/cm^3^)	1.0429		
°Brix	11.2	6.7	6.7
Total acidity reported as malic acid pH 7.0 (g/L)	5.6	3.7	3.7
Total acidity reported as citric acid pH 8.1 (g/L)	5.1	3.1	
Fructose (g/L)	58.4	35.0	35.0
Glucose (g/L)	22.4	13.4	13.4
Sucrose (g/L)	15.2	9.1	9.1
Total sugars (g/L)	95.9	57.5	57.5
Pectin (galacturonic acid mg/L)	114.0	68.4	
Potassium (mg/L)	990.0	590.0	590.0
Vitamin C (mg/L)	1.0	0.6	
Sorbitol (g/L)	3.7	2.2	2.2

### Study participants

This randomized controlled human intervention study in cross-over design was approved by the Ethics committee (reference number: 171/2021) of the German Sport University Cologne. The study was carried out between December 2021 and March 2022 in accordance with the ethical standards laid down in the Declaration of Helsinki. The study is registered at the German Clinical Trials Register (DRKS-ID00027860). All participants of the study were recruited in local sports groups and in courses of the German Sport University Cologne. Participants were healthy, normal weight (BMI > 18.5 and <24.9 kg/m²), non-smokers, and aged 18–35 years. None of the participants reported to drink more than the moderate amount of alcohol (<10 g/day for women; <20 g/day for men). Sample size was calculated using G*Power (Gpower, Version 3.1.9.2, Düsseldorf Germany). Herein, bacterial endotoxin levels were selected as primary endpoint. Sample size estimation was based on a one-way analysis of variance design with three groups and the assumption that the intake of the different beverages may alter bacterial endotoxin concentration in serum. Effect size was based on previously published data^21^. An effect size of 0.8, a power of 0.8 and an alpha level of 0.05 was used and revealed that 19 participants would be needed. Considering the above scenario and a drop-out rate of ~30% due to potential COVID-19 infections and a possible lack of compliance to the nutritional intervention, a total of 26 participants were enrolled in the study. A total of 19 participants completed the study. Participants dropped out of the study due to family reasons (*n* = 2), illness (*n* = 2) or a lack of compliance to the standardization of nutritional intake before the beverage challenge (*n* = 3).

### Intervention study

**Fig. 3:**
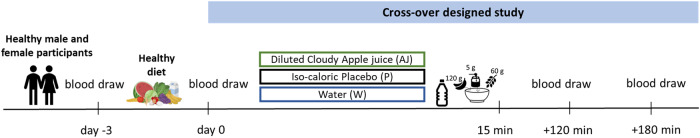
Study design of human intervention study. Parts of the graphical illustration were created with BioRender.com.

Before the study informed consent was obtained from all study participants and an initial health screen was conducted. Standardization of nutritional intake was carried out in accordance with Staltner et al.^21^. In brief, caloric intake of all participants was assessed in two separate 24-h recalls (one weekday and one weekend day). Prior to each intervention day, participants were asked to follow a standardized diet following the nutritional recommendations of the DACH nutritional societies being iso-caloric to their ‘normal’ diet for 3 days. All foods, beverages and recipes were provided by the study team. Nutritional intake and standardization of nutrition were calculated via the computer software EBISpro (Version 2011, Willstätt, Germany). The challenge with the different beverages was conducted in a cross-over randomized study design. Accordingly, participants were randomly assigned to the study interventions (block randomization via online calculator) as also summarized in Fig. 3. On the day of intervention (day 0) fasting blood was drawn before the participants received 500 mL of the respective study beverage and a light breakfast (60 g oatmeal containing 5 g honey prepared with water and a banana, for nutrition values of the breakfast please see Supplementary Table 2). The participants were asked to drink the respective beverage within 15 min in combination with the standardized breakfast. Additional blood samples were drawn 120 and 180 min after the consumption of the beverage. Each intervention day was followed by a wash-out period of at least one week.

### Analytical details of study beverages

#### Density and brix

The density was measured in apple juices by means of an oscillating U-tube (Anton Paar, Seelze, Germany). The corresponding Brix is calculated using the density table of the International Fruit and Vegetable Juice Association (IFU) no. 01a (Rev. 2005) and should correspond to the Brix corrected (Bx. Corr.)^56^.

#### Total acidity

The total acidity was determined titrimetrically according to IFU method no. 03 (Rev. 2017)^56^.

#### Spectrophotometric determination of the pectin content

Pectin was performed according to IFU method no. 26 (Rev. 2012)^56^.

#### Determination of the mineral content using Optical emission spectrometry with inductively coupled plasma (ICP-OES)

The mineral content was determined by ICP-OES after microwave digestion. The determination of minerals was carried out analogous to ASU method L.00.00-19/1. Prior to this, the samples were subjected to microwave digestion, which was carried out analogous to ASU method L.00.00-144^57^.

#### Enzymatic determination of the sorbitol content

The sorbitol content in the beverage was automatically determined enzymatically and kinetically using a test kit from Boehringer Mannheim/ r-biopharm (Darmstadt, Germany). With this method, the starting kinetics of the dye formation is determined. The slope is proportional to the substrate content.

#### Characterization and quantification of the sugar profile using high-performance liquid chromatography with refractive index detector (HPLC-RID)

The quantification of the sugars glucose, fructose, and sucrose was performed using HPLC-RID according to IFU^56^.

#### Determination of the vitamin C content using high-performance liquid chromatography with photodiode array (HPLC-DAD)

The quantification of vitamin C was performed using HPLC-DAD according to IFU method no. 17a (Rev. 2022)^56^.

#### Total phenolic content determined by Folin-Ciocalteu

To evaluate the total phenolic content the method according to Folin-Ciocalteau^55^ was used.

#### Qualitative and quantitative analysis of phenolic compounds using ultra-high-performance liquid chromatography with photodiode array (UHPLC-DAD)

The quantification of anthocyanins, phenolic acids and flavonols was performed on a Kinetex C18 (2.6 µm, 150 mm × 4.6 mm) column (Phenomenex, Aschaffenburg, Germany) using a 1290 Infinity II UHPLC system (Agilent, Waldbronn, Germany). Quantification was performed with the solvent using solvent systems A (0.1% formic acid) and B (acetonitrile) at a flow rate of 0.5 mL/min and 25 °C and the following gradient: 0 min 5% B, 40 min 95% B, 45 min 95% B, 50 min 5% B. Flavonols were detected at λ = 320 nm, phenolic acids at λ = 280 nm. The quantification was carried out as chlorogenic acid (5-CQA, 0.003–1 mg/mL, LOQ 0.04 mg/mL, LOD 0.001 mg/mL) as standard for phenolic acids, quercetin-3-O-glucoside (0.005–1.1 mg/mL, LOQ 0.14 mg/mL, LOD 0.004 mg/mL) as standard for flavonols.

### Anthropometry and metabolic parameters

Anthropometric data were determined at the beginning of the study. Baseline blood glucose levels were measured in serum of participants by using a glucometer as well as triglyceride levels were determined using a commercially available triglyceride kit (Pointe Scientific, Inc., Brussels, Belgium).

### Bacterial endotoxin and TLR2 ligands

Bacterial endotoxin levels and concentrations of TLR2 ligands were assessed in serum of participants as well as in media of cell culture experiments (see below) using SEAP reporter HEK 293 cell assays (Invivogen, Toulouse, France) activated by TLR2 and 4 ligands as detailed previously^58^.

### Enzyme-linked immunosorbent assay (ELISA)

Concentrations of i-FABP as well as sCd14 in serum of participants were determined using commercially available ELISA kits (Bio-Techne Corp., Minneapolis, MN, USA) according to the manufacturer.

### Caco-2 cells in vitro experiments

As described in detail previously^21^, Caco-2 cells (ACC 169, DSMZ, Braunschweig, Germany) were grown in trans-wells by using DMEM medium containing 10% fetal bovine serum (Pan-Biotech GmbH, Aidenbach, Germany) and 100 µg/mL streptomycin and 100 U/mL penicillin (Pan-Biotech GmbH, Aidenbach, Germany) in a 5% carbon dioxide atmosphere at 37 °C until confluency and differentiated for 9 days. On the day of experimentation, diluted cloudy apple juice or placebo (iso-sweet to the diluted cloudy apple juice, 0.06 g/mL total sugar: sucrose, fructose and glucose) was added to the apical compartment of the trans-wells for 2 h as shown in Fig. 2a. Thereafter, as also described in detail before^21^, LPS (100 ng/mL) and LTA (10 µg/mL), respectively, were added to the apical compartment for 1 h. Supernatant and media from the basolateral compartment were collected. Samples were stored at −80 °C until further analysis.

### Fructose measurement

Fructose concentration was measured in the basolateral compartment of the Caco-2 trans-well model using a commercially available Fructose Assay kit (Abnova GmbH, Heidelberg, Germany) after incubating cells with either diluted cloudy apple juice or placebo for 3 h.

### Statistical analysis

Data are presented as mean ± SEM. Normality of distribution of data was analyzed using Shapiro-Wilk normality test. Grubb’s test was performed before statistical analysis to identify outliers. To assess the effects between two groups an unpaired *t-*test was used. An ordinary one-way ANOVA with Dunnett’s multiple comparison test was used to determine statistically significant differences between interventions. All data were analyzed with GraphPad Prism software (GraphPad Prism Software Inc., San Diego, CA, USA). Significance was considered at a *p*-value < 0.05.

### Supplementary information

Supplementary Information

## Data Availability

The data presented in this study are available on request from the corresponding author due to legal and ethical reasons.
